# Effects of Initial Periodontal Therapy on Intraoral Bacterial Flora in Patients With Oral Contraceptive‐Related Severe Gingivitis

**DOI:** 10.1155/crid/5078642

**Published:** 2026-03-09

**Authors:** Aki Kawano, Keisuke Yamashiro, Yukiko Takahashi, Yukie Yoshida

**Affiliations:** ^1^ Department of Oral Health Sciences, Faculty of Health Sciences, Kobe Tokiwa University, Kobe, Hyogo, Japan

## Abstract

Gingivitis is a common inflammatory condition of the gingival tissues, influenced by microbial plaque and various systemic factors, including hormonal changes. Oral contraceptives containing estrogen and progestin are known to exacerbate gingival inflammation. We report the case of a 20‐year‐old Japanese woman who presented with gingival swelling and bleeding despite self‐care, with a history of orthodontic treatment and oral contraceptive use. Clinical examination revealed probing pocket depths of ≥ 4 mm and bleeding on probing in more than half of the sites, a periodontal inflamed surface area (PISA) of 995.8 mm^2^, and a plaque control record of 78%. Bacterial analysis identified elevated levels of *Prevotella intermedia*, *Prevotella melaninogenica*, and *Fusobacterium nucleatum*. Initial periodontal therapy—consisting of scaling and root planing, supported with systemic and topical antibiotics—was provided, followed by gingivectomy at sites lacking adequate gingival attachment. Posttreatment evaluation demonstrated marked reductions in probing depth, bleeding on probing, and PISA (44.9 mm^2^), with plaque control improving to 31.3%. Microbiome analysis revealed a decrease in pathogenic anaerobes and an increase in beneficial aerobic bacteria. The patient′s oral hygiene practices improved substantially through targeted education and self‐care instruction, supporting the maintenance of periodontal health. This case highlights that initial periodontal therapy, combined with patient education and regular monitoring, can effectively reduce gingival inflammation and favorably modify the oral microbiome in oral contraceptive‐related gingivitis. Awareness of the potential impact of oral contraceptives on periodontal health and implementation of individualized oral hygiene measures are essential for preventing disease progression in similar patients.

## 1. Introduction

Gingivitis is caused by substances derived from microbial plaque that accumulate within or near the gingival sulcus. Persistent gingivitis is a risk factor for periodontal attachment loss and, ultimately, tooth loss [1, 2]. Therefore, controlling gingival inflammation is essential to prevent progression to periodontal disease [3]. Gingivitis affects 50%–90% of individuals worldwide, and persistent gingival inflammation in young individuals is strongly associated with the development of periodontitis in adulthood [4]. In Japan, a 2016 national survey on dental disease reported that the prevalence of gingivitis (probing pocket depth of ≥ 4 mm) among individuals aged 15–24 years was 17.6%, and approximately 70% of adults aged 35–69 years exhibited signs of gingival disease [5].

The clinical expression of plaque‐induced gingival inflammation can be substantially modified by systemic factors that influence the host response [6], including diabetes [7, 8], cardiovascular disease [9], drug‐induced conditions [10], and nutritional deficiencies [11]. Hormonal changes during puberty, the menstrual cycle, pregnancy, menopause, and the use of oral or injectable contraceptives may also exacerbate plaque‐induced gingivitis.

Several studies have investigated the relationship between oral contraceptives and gingivitis [12, 13]. Although oral contraceptives are commonly used to treat irregular menstruation, they may also induce gingival inflammation [14]. The hormones contained in contraceptive pills, particularly estrogen and progesterone, have been suggested to influence gingival blood flow and immune responses, potentially worsening periodontal conditions [15]. This case is notable in that it demonstrates the clinical course of severe gingivitis in a young woman using oral contraceptives, incorporating clinical parameters and microbiome analysis. Unlike most previous reports, which primarily focused on clinical manifestations, this case integrates next‐generation sequencing–based microbial profiling to characterize bacterial changes following initial periodontal therapy. Furthermore, it highlights a potential link between hormone‐related modulation and alterations in the subgingival microbiome.

In this study, we aim to investigate the effects of initial periodontal therapy in patients with severe gingivitis associated with oral contraceptive use and to evaluate changes in the intraoral bacterial flora following treatment. We conducted a clinical study involving patients diagnosed with severe gingivitis who were using oral contraceptives. The patients underwent initial periodontal therapy, including scaling and root planing, over a period of more than 4 weeks. Clinical parameters of gingival inflammation, such as bleeding on probing (BOP) and probing pocket depth, were assessed before and after therapy. Additionally, intraoral bacterial samples were collected, and changes in bacterial flora were analyzed using molecular techniques. As part of this study, we include a representative case that integrates research findings and clinical practice.

## 2. Case Presentation

A preprint of this study has previously been published on Research Square [16]. The following case describes a patient with oral contraceptive‐related severe gingivitis who underwent initial periodontal therapy.

### 2.1. Patient Information

The patient was a 20‐year‐old Japanese woman who presented with gingival swelling and bleeding during toothbrushing in July 2022. Because her symptoms did not resolve, she sought care at our clinic. Her medical history included chronic sinusitis and orthodontic treatment from 2017 to 2018.

She began taking oral contraceptives in 2021 for irregular menstruation and initially experienced adverse effects, including nausea and irregular bleeding. These events resolved after consultation with a geneticist and switching medications in April 2022, after which her menstrual irregularity improved. Her current oral contraceptive contained desogestrel 0.05 mg and ethinylestradiol 0.03 mg. She was a nonsmoker, had no systemic diseases, and had not taken antibiotics within the preceding 6 months.

### 2.2. Clinical Oral Examinations

A full‐mouth periodontal examination, including radiographic assessment, was performed. The periodontal inflamed surface area (PISA) was calculated based on periodontal pocket depth (PPD) and BOP at six sites per tooth. PISA represents the surface area (mm^2^) of the bleeding pocket epithelium [17]. Tooth mobility was evaluated using the Miller mobility index, and plaque accumulation was measured using the O′Leary plaque control record (PCR).

### 2.3. Differential Diagnosis, Investigations, and Treatment

The patient was diagnosed with a severe plaque‐induced gingivitis. Because of her long‐term oral contraceptive use, a bacterial test was performed at the first visit. Initial periodontal therapy, consisting of plaque control instruction, scaling, and antimicrobial therapy, was initiated. Minocycline was used as a topical agent, and azithromycin was prescribed systemically. According to the clinical practice guidelines of the Japanese Society of Periodontology, a sustained‐release tetracycline ointment is recommended as the first‐line local antimicrobial therapy for periodontal pockets. Therefore, minocycline hydrochloride (10 mg, 0.5 g per pocket) was selected. Minocycline achieves high local concentrations and effectively suppresses obligate anaerobic periodontopathogenic bacteria in deep pockets.

Because the inflammatory response was insufficiently controlled with initial therapy alone, systemic azithromycin (Zithromax 250 mg, two tablets once daily for three consecutive days) was added. Azithromycin was selected because of its antimicrobial activity against periodontopathogens and favorable pharmacokinetic properties, including high tissue penetration, long intracellular half‐life enabling short‐course dosing, and host‐modulatory effects such as reduction of pro‐inflammatory cytokines and suppression of biofilm formation. These characteristics support its clinical use for refractory periodontal inflammation. After completion of initial therapy, a follow‐up bacterial test was performed. Re‐evaluation showed improvement in gingival redness and swelling; however, the mandibular anterior region lacked gingival attachment, necessitating gingivectomy. Following the procedure, the patient was re‐evaluated and transitioned to supportive periodontal therapy (SPT). The overall treatment timeline—from the initial examination in December 2022 to re‐evaluation in April 2023 and transition to SPT in October 2023—followed the standard clinical sequence recommended in periodontal treatment guidelines (initial therapy → re‐evaluation → SPT), with shortened visit intervals and surgical intervention added according to the patient′s clinical needs.

### 2.4. Extraction of Periodontal Bacterial DNA and Microbiome Analysis

Gingival crevice fluid samples were collected from the deepest periodontal pockets using three sterile paper points inserted into each site for 1 min. Deep pockets were selected because they provide anaerobic conditions favorable for periodontopathic bacteria. Sampling from shallow sites (≤ 3 mm) may not accurately represent the microbiome of active disease because of higher oxygen exposure. An amplicon library was constructed from each bacterial DNA sample using polymerase chain reaction–based amplification targeting the hypervariable V3–V4 regions of the 16S rRNA gene. Illumina adapter overhang nucleotide sequences were added, and polymerase chain reaction was performed according to the manufacturer′s protocol (Illumina, San Diego, California, United States). Next‐generation sequencing was conducted using the MiSeq system (Illumina) at the Oral Microbiome Center (Kagawa, Japan). Sequence analysis was performed using QIIME2 according to the online tutorial (https://view.qiime2.org/), and the obtained sequences were compared with the expanded Human Oral Microbiome Database (https://www.homd.org/).

### 2.5. Taking Oral Contraceptives and Poor Plaque Control

At the initial examination, the patient exhibited gingival swelling, redness, and substantial plaque accumulation. Dehiscence and lack of gingival attachment were noted on the labial aspect of the mandibular anterior teeth (Figure 1a). Radiographic evaluation confirmed the absence of alveolar bone resorption (Figure 1b). Table 1 summarizes the periodontal findings. At baseline, a PPD of ≥ 4 mm was present in half of the dentition (normal: ≤ 3 mm). Widespread inflammation was evident, with a BOP of 49.4% (clinical threshold for active inflammation: 20%). The PISA was 995.8 mm^2^, indicating a marked gingival inflammation despite no radiographic bone loss. Previous studies have reported that moderate to severe periodontitis with an initial PISA of approximately 1500 mm^2^ can be reduced to < 100 mm^2^ after SPT. In this case, therapy reduced the PISA to 44.9 mm^2^, an improvement comparable with outcomes reported in the literature. The patient′s oral hygiene status was poor at baseline, with a PCR value of 78% (good: < 20%; fair: 21%–40%; poor: > 40%).

Figure 1Clinical and radiographic findings at the first visit. (a) Intraoral photographs showing widespread gingival redness and swelling, with dehiscence observed on the labial aspect of the mandibular anterior teeth. (b) Panoramic radiograph demonstrating overall alveolar bone levels at the first visit.

**Figure:**
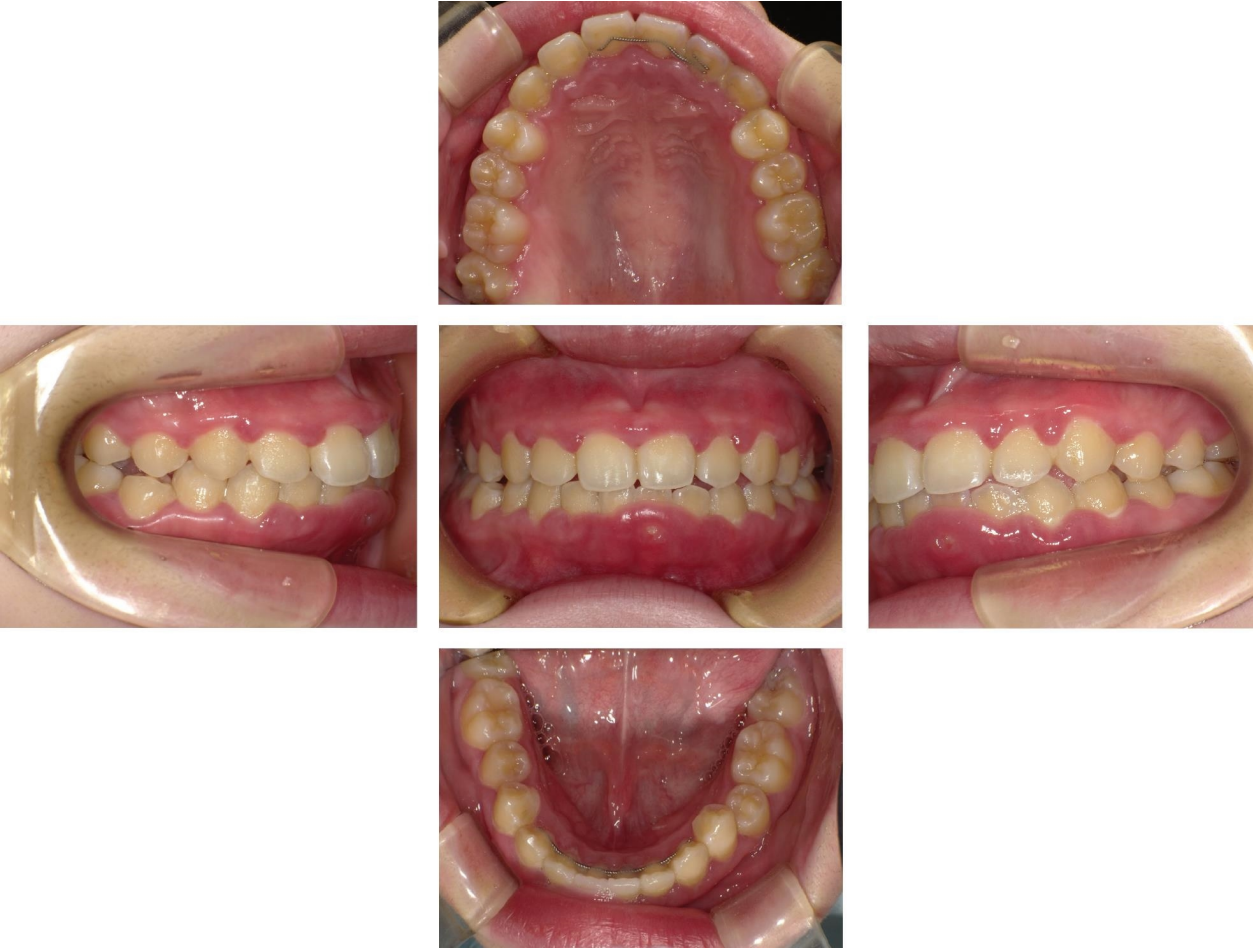
(a)

**Figure:**
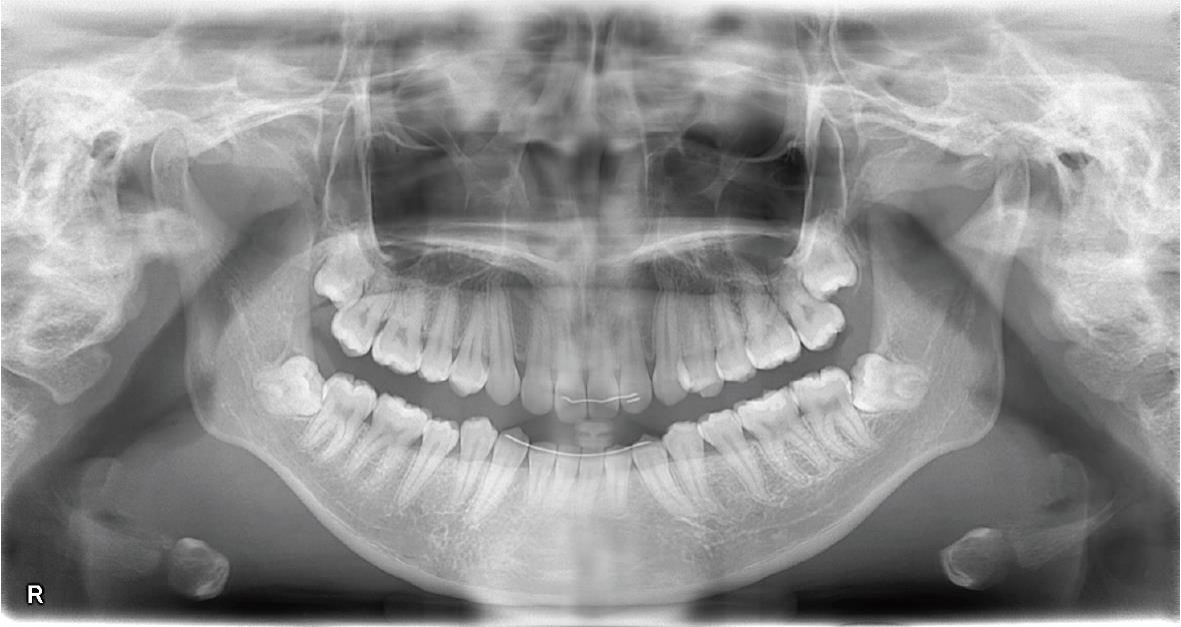
(b)

**Table 1:** Clinical parameters for periodontal diagnosis and locations of sampling sites.

Data	Total teeth (*n*)	BOP (%)	Mean PPD (mm)	1–3 mm PPD (%)	4–5 mm PPD (%)	≥ 6 mmPPD (%)	PISA (mm^2^)	PESA (mm^2^)	PCR (%)
**December 12, 2022**	28	49.4	3.5	50.0	47.0	3.0	995.8	2,037.2	89.3
**April 19, 2023**	28	8.3	2	98.2	1.8	0	76.0	1,082.6	75.9
**October 4, 2023**	28	6.5	1.7	100	0	0	44.9	917.6	31.3

*Note:* The initial examination was performed in December 2022, basic periodontal treatment was completed in April 2023, and the patient transitioned to SPT in October 2023.

Abbreviations: BOP, bleeding on probing; PCR, plaque control record; PESA, periodontal epithelial surface area; PISA, periodontal inflamed surface area; PPD, periodontal pocket depth; SPT, supportive periodontal therapy.

### 2.6. Dental Hygiene Interventions and Patient Awareness

Dental hygienist interventions were performed at three key stages: the initial consultation, the completion of initial therapy, and the transition to SPT. At the first visit, the patient reported being unable to brush adequately because of pain and bleeding. PISA measured 995.8 mm^2^ (Table 1), and severe inflammation was present. She received instruction on proper brushing technique using an extra–super‐soft toothbrush and a plaque‐disclosing agent. Education was also provided regarding the effects of oral contraceptives on gingival health, as her understanding of medication‐related gingival changes was limited.

**Figure 2:**
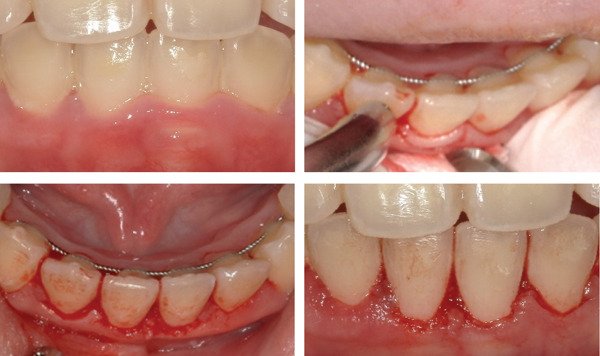
Intraoperative photographs during gingivectomy. Gingival tissue lacking attachment was excised from the mandibular anterior region under local anesthesia.

Initial periodontal therapy and topical antibiotic application resulted in partial improvement; however, persistent lack of attachment in the mandibular anterior region necessitated gingivectomy (Figure 2). Postoperatively, gingival appearance and attachment improved, and the patient′s motivation for oral hygiene increased. She continued self‐care using a plaque‐disclosing agent.

**Figure 3:**
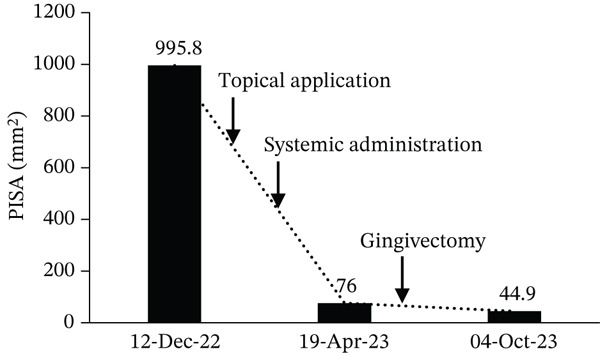
Changes in periodontal inflamed surface area (PISA) over the course of treatment. PISA values are shown at the first visit, after antibiotic administration, and after completion of initial periodontal therapy. A marked reduction in PISA was observed, indicating substantial improvement in gingival inflammation.

**Figure 4:**
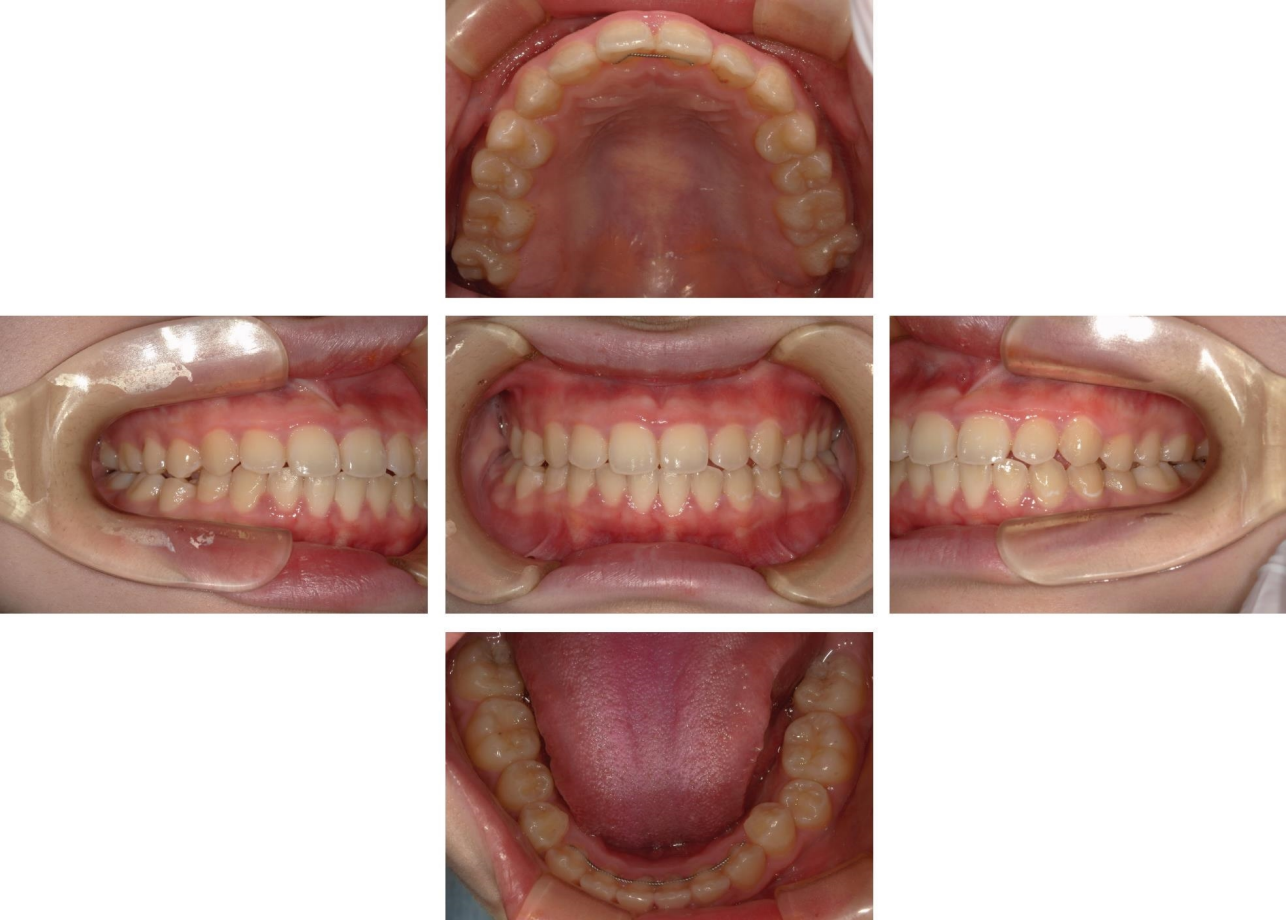
Intraoral photographs at the transition to supportive periodontal therapy. Inflammation and attachment were improved, with no redness or swelling observed.

The mesial‐buccal sites of Teeth 31 and 41, which initially exhibited the deepest pockets, improved from 6 mm at baseline to 4 mm after initial therapy and to 2 mm at the transition to SPT (data not shown). PISA decreased from 995.8 mm^2^ to 44.9 mm^2^ at the transition to SPT (Figure 3). With continued dental hygiene support, PCR improved from 89.3% to 31.3% (Table 1 and Figure 4).

### 2.7. Changes in Oral Bacterial Flora Following Initial Periodontal Therapy

**Figure 5:**
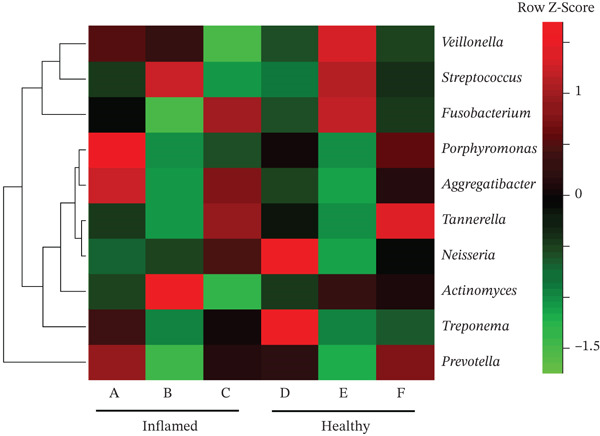
Heatmap of bacterial flora in inflamed and healthy sites. The inflamed site was the mesial‐buccal aspect of Tooth 41: (A) first visit, (B) after antibiotic administration, and (C) after initial periodontal therapy. The healthy control site was the distal‐buccal aspect of Tooth 42: (D) first visit, (E) after antibiotic administration, and (F) after initial periodontal therapy. Color transition from green to red indicates increasing relative abundance.

Obligate anaerobes, including *Fusobacterium*, *Porphyromonas*, and *Prevotella*, were detected at the inflamed site during the initial examination (Figure 5). In contrast, the control site showed no obligate anaerobes but contained aerobic species, such as *Neisseria* and *Actinomyces*. Among the *Prevotella* species, *Prevotella intermedia* and *Prevotella melaninogenica* were abundant at the inflamed site and were successfully suppressed following antibiotic therapy and initial treatment. *Fusobacterium nucleatum* decreased after antibiotic administration but increased again at the end of initial treatment (Table 2 and Figure 6).

**Table 2:** Occupancy of bacterial species in inflamed and healthy sites.

Bacterial species	Site	First visit (%)	After administration (%)	After basic periodontal therapy (%)
*Fusobacterium nucleatum*	Inflamed	0.0442	N.D.	0.0436
	Healthy	0.0154	N.D.	0.0004
*Porphyromonas endodontalis*	Inflamed	0.0372	0.0001	0.0001
	Healthy	0.0114	0.0001	N.D.
*Prevotella intermedia*	Inflamed	0.0072	0.0004	N.D.
	Healthy	0.0005	N.D.	N.D.
*Prevotella melaninogenica*	Inflamed	0.1374	0.0192	0.0033
	Healthy	0.0535	0.0089	0.0492
*Streptococcus intermedius*	Inflamed	0.0264	0.0049	0.0006
	Healthy	0.0049	0.0038	0.0013

The inflamed site was the mesial‐buccal aspect of Tooth 41, and the healthy site was the distal‐buccal aspect of Tooth 42. Sample sites were numbered according to the FDI World Dental Federation notation (e.g., 41, 42).

Abbreviation: N.D., not detected (detection threshold: < 0.01% relative abundance).

Figure 6Changes in the relative abundance of selected bacterial species over the course of treatment. The proportions of selected bacterial species are shown at three time points: first visit, after antibiotic administration, and after completion of initial periodontal therapy. Data are presented separately for inflamed and healthy sites. (a) *Fusobacterium nucleatum*, (b) *Porphyromonas endodontalis*, (c) *Prevotella intermedia*, (d) *Prevotella melaninogenica*, (e) *Streptococcus intermedius*.

**Figure:**
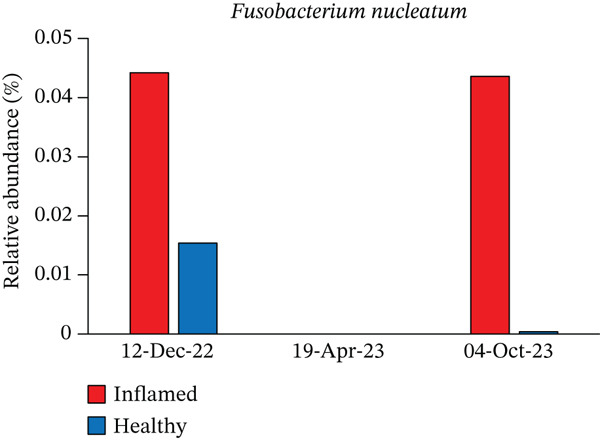
(a)

**Figure:**
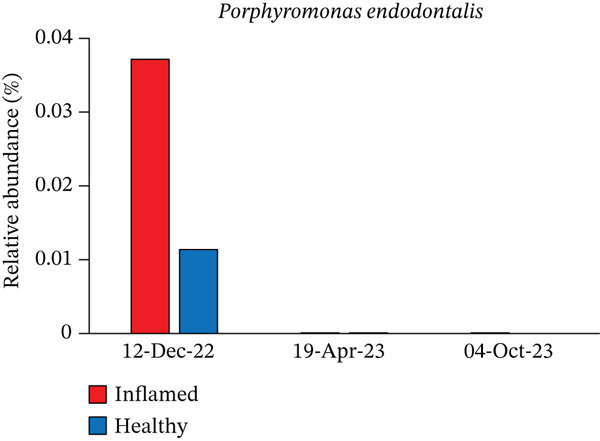
(b)

**Figure:**
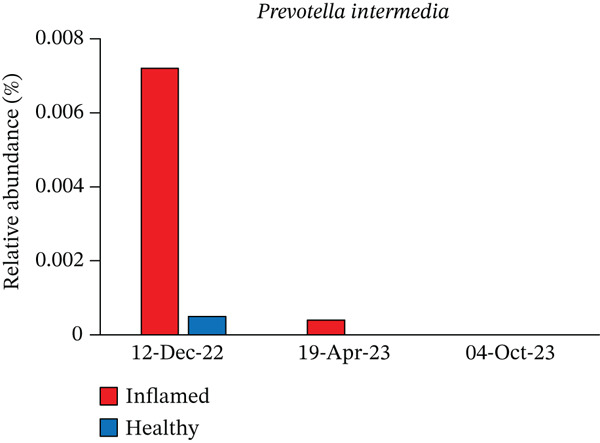
(c)

**Figure:**
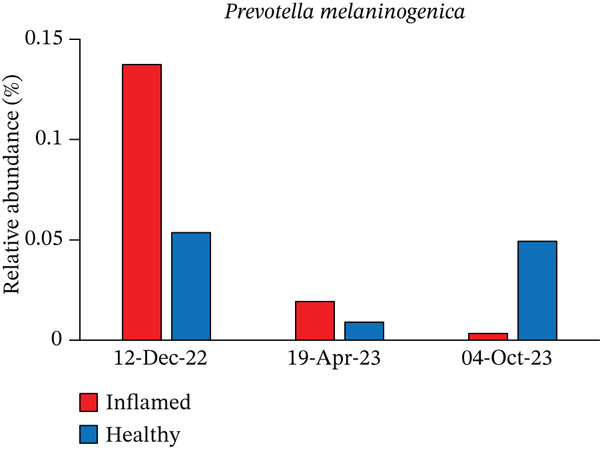
(d)

**Figure:**
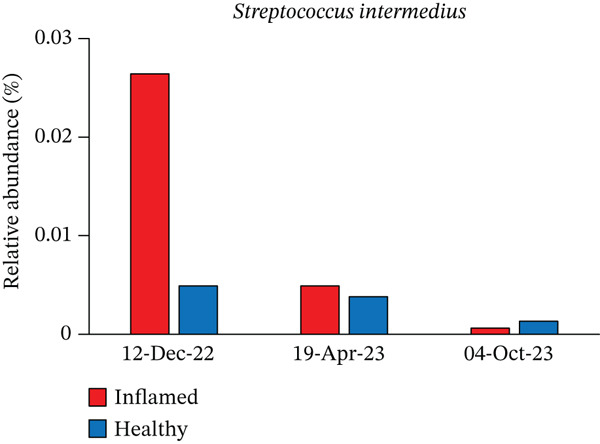
(e)

## 3. Discussion

Our results demonstrated a significant reduction in gingival inflammation following initial periodontal therapy. Beyond summarizing these clinical improvements, it is important to consider the underlying biological mechanisms. The marked changes in BOP, PPD, and subgingival microbiota suggest that microbial and host‐modulatory pathways were altered through treatment.

The microbiological analysis revealed notable shifts in subgingival bacterial composition, particularly among hormonally responsive species. Among the 103 genera analyzed, *Prevotella*—and specifically *P*. *intermedia*—showed the most pronounced reduction. *P. intermedia* is known to increase protein expression and proliferation in the presence of estradiol and progesterone [18]. Because the patient was taking a combination oral contraceptive containing desogestrel and ethinylestradiol, these hormones may have contributed to the exacerbation of the initial gingival inflammation. Previous studies, including the report by Brusca et al. [12], likewise demonstrated that oral contraceptive use can alter the host environment by increasing vascular permeability and modifying subgingival microbial profiles. In contrast to Brusca′s cross‐sectional findings, our case provides longitudinal evidence that hormonally associated microbial changes can be reversed through appropriate periodontal therapy.

Mechanistically, estrogen and progesterone exert multiple effects on periodontal tissues. Both hormones enhance vascular permeability and capillary proliferation, resulting in exaggerated inflammatory responses to dental plaque [19, 20]. Additionally, they modulate immune and host‐defense mechanisms. Estrogen may influence the production of pro‐ and anti‐inflammatory cytokines, whereas progesterone can alter neutrophil chemotaxis and phagocytic activity, ultimately affecting the host′s susceptibility to periodontal tissue changes [21, 22]. Therefore, the reduction in *Prevotella* observed after therapy likely reflects improved plaque control and the normalization of a hormone‐modified subgingival environment.

The combined use of topical minocycline and systemic azithromycin contributed to the rapid improvements in clinical and microbiological parameters. Local minocycline provides high drug concentrations directly within periodontal pockets, though its therapeutic reach and duration are limited. Systemic azithromycin, in contrast, offers deep tissue penetration, a long intracellular half‐life that supports short‐course dosing, and host‐modulatory anti‐inflammatory effects. However, systemic antibiotics also present disadvantages, including the potential for antimicrobial resistance, gastrointestinal side effects, and microbiome disruption. These considerations highlight the need for individualized treatment planning, particularly in hormonally influenced gingival conditions.

This report describes a single case, and the follow‐up period was limited. The uniqueness of the case and the interindividual variability of the oral microbiome limit the generalizability of the findings. Larger studies with longitudinal microbiome assessments are needed to clarify the influence of oral contraceptives on periodontal health. Future research may explore natural therapeutic agents such as probiotics, paraprobiotics, postbiotics, and ozonated compounds [23], as well as adjunctive technologies such as laser therapy for managing gingival inflammation [24].

In conclusion, initial periodontal therapy effectively reduced gingival inflammation associated with oral contraceptive use, as demonstrated by marked reductions in PISA and improvements in the subgingival microbiota. These findings suggest that targeted periodontal interventions, combining local and systemic antibacterial strategies, can help restore microbial balance and support long‐term periodontal stability. Proactive oral hygiene instruction and regular monitoring are recommended for patients receiving hormonal contraceptives to prevent disease recurrence.

## Author Contributions

A.K., K.Y., Y.T., and Y.Y. performed the experiments. A.K. drafted the initial version of the manuscript, and K.Y. assisted with manuscript preparation. A.K., K.Y., Y.T., and Y.Y. designed the experiments.

## Funding

This work was supported by the Japan Society for the Promotion of Science through a Grant‐in‐Aid for Scientific Research (KAKENHI) (Grant Number 21K17207).

## Disclosure

All authors contributed to data collection and interpretation, critically reviewed the manuscript, and approved the final version.

## Ethics Statement

This case study was conducted in accordance with the guidelines of Kobe Tokiwa University, and written informed consent was obtained from the patient. Written informed consent was also obtained from the patient for the publication of this case report and any accompanying images.

## Conflicts of Interest

The authors declare no conflicts of interest.

## Data Availability

The clinical data supporting this case report are not publicly available in order to protect patient privacy and confidentiality. De‐identified data relevant to this case may be obtained from the corresponding author upon reasonable request. All literature cited in this report is publicly accessible through the referenced journals and databases.
